# Supplementary material for: Identification of photoperiod-induced specific miRNAs in the adrenal glands of Sunite sheep (Ovis aries)
Source: Front Vet Sci. 2022 Jul 22;9:888207. doi: 10.3389/fvets.2022.888207 (PMC9354845; doi:10.3389/fvets.2022.888207)
Supplement: Supplementary file 1 [file Data_Sheet_1.ZIP › Supplementary file/miRDeep2 file.zip!/miRDeep2 file/Novel_Structure/SP42A3/Novel_179.pdf]

Provisional ID	:	NC_019459.2_3536
Score total	:	0.6
Score for star read(s)	:	-1.3
Score for read counts	:	-1.3
Score for mfe	:	1.7
Score for randfold	:	1.6
Score for cons. seed	:	
Total read count	:	9
Mature read count	:	9
Loop read count	:	0
Star read count	:	0

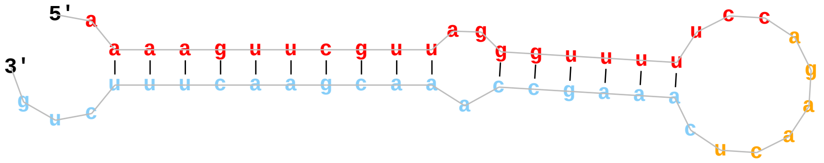

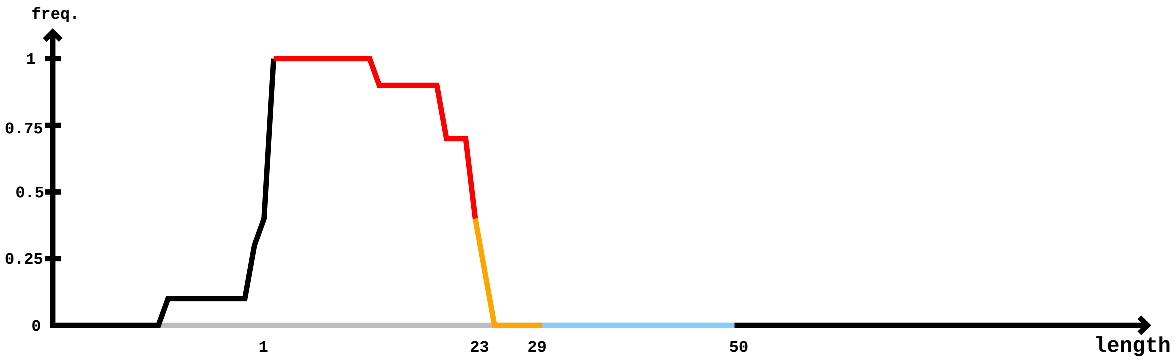

Mature Star

```
5'- uugcaaguauuggguuggccaaaaaguucguuaggguuuuuccagaacucaaagccaaacgaacuuucuggcuuaaccaauacuucauaaguguuagucgcucggucgugug  -3'   exp
    .((.(((((((((...(((((.((((((((((..((((((.........)))))).))))))))))).)))))...)))))))))))..((((.....))))..........    reads   mm   sample
    ..........uggguCggccaaaaaguucguu................................................................................    1       1    seq
    ...................caaaaaguucguuUggguuu.........................................................................    2       1    seq
    ....................aaaaaguucguuaggguuuuuc......................................................................    1       0    seq
    .....................aaaaguucAuuaggguuuuuc......................................................................    2       1    seq
    .....................aaaaguucAuuaggguuuuucc.....................................................................    4       1    seq
```
